# Special Issue: “Genomics of Stroke”

**DOI:** 10.3390/genes13030415

**Published:** 2022-02-25

**Authors:** Svetlana A. Limborska, Ivan B. Filippenkov

**Affiliations:** Institute of Molecular Genetics of National Research Center “Kurchatov Institute”, Kurchatov Sq. 2, 123182 Moscow, Russia; limbor@img.msk.ru

Stroke is a multifactorial disease and an extremely serious and socially important medical condition [1,2]. A recent economic analysis in Europe reported that stroke cost the area EUR 60 billion, with health care accounting for EUR 27 billion (45%), representing 1.7% of health expenditure [3]. The molecular genetic mechanisms for the pathogenesis and pharmacological correction of stroke remain largely unknown. However, the common use of high-throughput technologies, including genome-wide association studies (GWAS), DNA and RNA sequencing, and the development and analysis of model systems, has revealed genomic data suspected of being involved that may be used in predicting the risk and improving the diagnostics and treatment of stroke.

This Special Issue (SI) entitled “Genomics of Stroke” provides a platform for a wide range of papers related to genetic studies of the pathogenesis, progression, diagnosis, and treatment of stroke. This SI includes six research articles and four reviews.

The research articles in our SI are focused on association and functional studies of stroke-related genes or noncoding RNAs, and model systems, as well as pharmacogenomics and bioinformatics studies of stroke.

First, we consider a paper entitled “MicroRNA Analysis of Human Stroke Brain Tissue Resected during Decompressive Craniectomy/Stroke-Ectomy Surgery” by Carlson et al. It should be noted that the authors conducted a comprehensive study. Specifically, human brain samples were obtained during craniectomy and brain tissue resection in patients with severe stroke who had life-threatening brain swelling. The tissue samples were subjected to histopathological and immunofluorescence microscopy evaluation, next-generation miRNA sequencing (NGS), and bioinformatic analysis [4]. As a result, miRNA NGS analysis detected 34 miRNAs with significantly aberrant expression in stroke tissue, compared with non-stroke samples [4]. The authors concluded that dysregulated miRNAs detected in their study could be regarded as potential candidates for biomarkers and/or targets for therapeutic intervention [4].

The role of another type of regulatory RNA—namely, circular RNAs (circRNAs), in cerebral ischemia conditions was studied by Filippenkov et al., as reported in their paper entitled “Genome-Wide RNA-Sequencing Reveals Massive Circular RNA Expression Changes of the Neurotransmission Genes in the Rat Brain after Ischemia–Reperfusion”. This type of RNA remains a highly promising target for research. Filippenkov et al. conducted a genome-wide RNA-Seq analysis of the subcortical structures of the rat brain containing an ischemic damage focus and penumbra [5]. They found 395 circRNAs that changed their expression significantly at 24 h after transient middle cerebral artery occlusion (tMCAO). Furthermore, functional annotation revealed their association with neuroactive signaling pathways. They found that about a third of the differentially expressed circRNAs (DECs) originated from genes whose mRNA levels also changed at 24 h after tMCAO. The other DECs originated from genes encoding nonregulated mRNAs under tMCAO conditions. Moreover, the bioinformatic analysis predicted a circRNA–miRNA–mRNA network that is associated with neurotransmission signaling regulation [5]. Filippenkov et al. showed that such circRNAs can persist as potential miRNA sponges to protect mRNAs of neurotransmitter genes [5].

Interesting results were also obtained by Khrunin et al. in their paper entitled “Examination of Genetic Variants Revealed from a Rat Model of Brain Ischemia in Patients with Ischemic Stroke: A Pilot Study”. The authors were able to correlate the results of gene expression obtained in a rat model of tMCAO with the genomic characteristics of patients with ischemic stroke (IS). Previously, the authors had developed a bioinformatic approach to exploring single-nucleotide polymorphisms (SNPs) in human orthologues of rat genes expressed differentially under conditions of induced brain ischemia [6]. Using this approach, the authors identified and analyzed nine SNPs in 553 Russian individuals (331 patients with IS and 222 controls) [7]. Moreover, Khrunin et al. explored the association of SNPs with both IS outcomes and the risk of IS. SNP rs66782529 (LGALS3) was associated with negative IS outcomes (*p* = 0.048). SNPs rs62278647 and rs2316710 (PTX3) were associated significantly with IS (*p* = 0.000029 and *p* = 0.0025, respectively). These correlations for rs62278647 and rs2316710 were found only in women, which suggests a sex-specific association of the PTX3 polymorphism [7]. Thus, the research of Khrunin et al. does not only reveals some new genetic associations with IS and its outcomes but also shows how exploring variations in genes from a rat model of brain ischemia can be of use in searching for human genetic markers of this disorder [7].

Lee et al. present their paper entitled “Association of CYP26C1 Promoter Hypomethylation with Small Vessel Occlusion in Korean Subjects” related to epigenetic studies of stroke. In their study, the authors measured the level of DNA methylation in the CYP26C1 promoter and the 5′ untranslated region of 115 normal subjects and 56 patients in Korea with small-vessel occlusion (SVO) [8]. They found an association between hypomethylation in this region and SVO in elderly Korean subjects. The authors also identified an association between methylation at specific CpG sites in the promoter region of cytochrome P450 family 26 subfamily C member 1 (CYP26C1) and blood parameters related to SVO [8]. Furthermore, they showed that retinoid X receptor RXR-α and retinoic acid receptor RAR-β might be affected by CYP26C1 methylation. From these findings, the authors concluded that CYP26C1 methylation may be an epigenetic potential indicator of SVO [8].

Tanaka et al. present their paper entitled “Influence of Renal Impairment and Genetic Subtypes on Warfarin Control in Japanese Patients”, which refers to pharmacogenomic research. It is known that warfarin is an effective means of anticoagulant therapy. However, warfarin has a narrow therapeutic range and shows a wide variation in doses between patients because of numerous environmental and genetic factors that influence warfarin pharmacokinetics and pharmacodynamics. Moreover, the genotypes of vitamin K epoxide reductase complex 1 (VKORC1) and cytochrome P450 2C9 (CYP2C9) can influence therapeutic warfarin doses [9,10,11,12]. The variant CYP2C9 and wild-type VKORC1, which affect the action of warfarin, are relatively rare in the Japanese population. Tanaka et al. enrolled 176 Japanese outpatients who were prescribed warfarin for thromboembolic stroke prophylaxis in the stroke center in their study. Patient characteristics, blood test results, dietary vitamin K intake, and CYP2C9 and VKORC1 (−1639G>A) genotypes were recorded [9]. CYP2C9 and VKORC1 (−1639G>A) genotyping revealed that 80% of the patients had CYP2C9 *1/*1 and VKORC1 mutant AA genotypes. Multiple linear regression analysis demonstrated that the optimal pharmacogenetics-based model comprised age, body surface area, estimated glomerular filtration rate (eGFR), genotypes, vitamin K intake, aspartate aminotransferase levels, and alcohol intake [9].

Finally, our SI includes an interesting research article by Leira et al. based on the application of bioinformatics methods entitled “Network Protein Interaction in the Link between Stroke and Periodontitis Interplay: A Pilot Bioinformatic Analysis”. In this exploratory study, the authors conducted a protein–protein network interaction (PPI) search with documented encoded proteins for both stroke and periodontitis [13]. Genes of interest were collected via a GWAS database. The STRING database was used to predict the PPI networks, first with a sensitivity purpose (confidence cutoff of 0.7) and then with the highest confidence cutoff (0.9). Gene overrepresentation was inspected in the final network. Leira et al. foresee a prospective protein network of interaction between stroke and periodontitis. Inflammation, procoagulant/prothrombotic state, and, ultimately, atheroma plaque rupture comprise the main biological mechanism derived from the network [13]. The authors conclude that these pilot results may shed light on future molecular and therapeutic studies to understand better the association between these two conditions [13].

Additionally, results and prospects for stroke genomics were systematized and discussed in substantial reviews.

The first review in our SI entitled “Monogenic Causes of Strokes” by Chojdak-Łukasiewicz et al. reports that monogenic disorders are rare but play significant roles in the causes of strokes, especially in young people. The monogenic causes of stroke are recognizable by key clinical features and radiographic pictures. Genetic tests are expensive but should be part of routine diagnostic procedures in younger patients with cerebrovascular events, especially in the absence of typical vascular risk factors [14]. The most common single-gene diseases connected with strokes are cerebral autosomal dominant arteriopathy with subcortical infarcts and leukoencephalopathy (CADASIL); Fabry disease; mitochondrial myopathy, encephalopathy, lactacidosis, and stroke (MELAS); many single-gene diseases associated particularly with the cerebral small-vessel disease, such as COL4A1 syndrome, cerebral autosomal recessive arteriopathy with subcortical infarcts and leukoencephalopathy (CARASIL), and hereditary endotheliopathy with retinopathy, nephropathy, and stroke (HERNS). In this review, the clinical phenotypes for the most important single-gene disorders associated with strokes are presented [14]. The authors conclude that early diagnosis of the monogenic causes of stroke is important to provide appropriate therapy when available [14].

The SI also includes a review characterizing one of the examples of ethnic features of stroke genomics. In their review entitled “Genetic and Genomic Epidemiology of Stroke in People of African Ancestry”, Prapiadou et al. describe significant racial/ethnic differences in the incidence, subtype, and prognosis of stroke between people of European and African ancestry, of which only about 50% can be explained by traditional stroke risk factors [15]. However, only a small number of genetic studies include individuals of African descent, leaving many gaps in our understanding of stroke genetics for this population [15]. This review highlights the need for, and significance of, including individuals with African ancestry in stroke genetic studies and points to the efforts that have been made toward this direction. Additionally, Prapiadou et al. discuss the caveats, opportunities, and next steps in stroke genetics of Africans—a field still in its infancy but with great potential for expanding our understanding of stroke biology and for developing new therapeutic strategies [15].

Another review entitled “The Genetic Landscape of Patent Foramen Ovale: A Systematic Review” by Paolucci et al. will undoubtedly attract attention to our SI. It should be noted that paradoxical embolism through patent foramen ovale (PFO) is an important cause of cryptogenic IS, especially in younger patients. Consistent with this observation, the rate of PFO in siblings of young patients with IS and PFO is three times higher than in siblings of patients without PFO [16,17,18]. In their review, Paolucci et al. evaluated the contribution of genetic alterations in PFO development [16]. Therefore, among 1231 studies searched, the authors included four: two of them assessed the NKX2-5 gene, and the remaining two reported variants of chromosome 4q25 and the GATA4 S377G variant, respectively. Paolucci et al. did not find any variant associated with PFO, except for the rs2200733 variant of chromosome 4q25 in patients with atrial fibrillation [16]. Further insight from genetic studies may help to evaluate better at the single-patient level the incidental or cocausative role of PFO in the setting of cryptogenic stroke, potential correlation with migraine, and potential value for genetic screening of at-risk first-degree relatives [16].

Finally, the review entitled “Influence of Haptoglobin Polymorphism on Stroke in Sickle Cell Disease Patients” by Edwards et al. is of substantial interest for our SI. This review outlines the current clinical research investigating how the haptoglobin (Hp) genetic polymorphism and stroke occurrence are implicated in sickle cell disease (SCD) pathophysiology [19]. Hp is an acute-phase protein capable of binding hemoglobin, thus preventing iron loss and renal damage [20]. Edwards et al. report the role of Hp in patients with SCD is critical in combating blood toxicity, inflammation, oxidative stress, and stroke [19]. IS occurs when a blocked vessel decreases blood-oxygen delivery to cerebral tissue and is commonly associated with SCD. Due to the malformed red blood cells resulting from sickle hemoglobin S, blockage of blood flow is significantly more prevalent in patients with SCD [19].

In conclusion, the diversity and quality of the studies presented in this SI indicate the constant advancement of knowledge in the field of genomics of stroke.

We hope that our SI will be useful and interesting for readers. Further development of the field of stroke genomics will bring society closer to improving diagnostic, prognostic, and therapeutic measures to combat stroke and related pathologies.
